# The journey of decellularized vessel: from laboratory to operating room

**DOI:** 10.3389/fbioe.2024.1413518

**Published:** 2024-06-25

**Authors:** Chenbin Kang, Hongji Yang

**Affiliations:** ^1^ School of Medicine, University of Electronic Science and Technology of China, Chengdu, China; ^2^ Organ Transplant Center, Sichuan Academy of Medical Sciences and Sichuan Provincial People’s Hospital, University of Electronic Science and Technology of China, Chengdu, China; ^3^ Clinical Immunology Translational Medicine Key Laboratory of Sichuan Province and Organ Transplantation Center, Sichuan Provincial People’s Hospital, University of Electronic Science and Technology of China, Chengdu, China

**Keywords:** decellularization, vessel, bioengineer, xenotransplantation, extracellular matrix

## Abstract

Over the past few decades, there has been a remarkable advancement in the field of transplantation. But the shortage of donors is still an urgent problem that requires immediate attention. As with xenotransplantation, bioengineered organs are promising solutions to the current shortage situation. And decellularization is a unique technology in organ-bioengineering. However, at present, there is no unified decellularization method for different tissues, and there is no gold-standard for evaluating decellularization efficiency. Meanwhile, recellularization, re-endothelialization and modification are needed to form transplantable organs. With this mind, we can start with decellularization and re-endothelialization or modification of small blood vessels, which would serve to address the shortage of small-diameter vessels while simultaneously gathering the requisite data and inspiration for further recellularization of the whole organ-scale vascular network. In this review, we collect the related experiments of decellularization and post-decellularization approaches of small vessels in recent years. Subsequently, we summarize the experience in relation to the decellularization and post-decellularization combinations, and put forward obstacle we face and possible solutions.

## 1 Introduction

### 1.1 The status of decellularized vessel

In 2020, there was 116,577 patients on the wait-list of organ transplantation in USA. (Sykes and Sachs, 2022) The situation that supply exceeds demand is similar with the field of vascular transplantation. Apart from haemodialysis, peripheral arterial diseases, venous thrombosis and trauma, which result in high morbidity and mortality (van der Velden et al., 2022), vascular substitutes are also urgently needed in living donor liver transplantation (Jeng et al., 2015; Sevmiş et al., 2023) and cardiovascular diseases. The use of large vessel substitutes by surgeons has been demonstrated to be a successful approach. However, there is still a need for alternatives for small blood vessels (internal diameter ≤ 6 mm). In order to minimize the gap, several strategies have been proposed. These strategies include: 1) Expanding the source of allogeneic donors (Truby et al., 2022); 2) Xenotransplantation; 3) Bioengineered organ.

The first method is also a more mature method at present, but it is plagued by organ quality, pathogen contamination and postoperative complications such as rejection, infection. Expanding the donor pool still does not solve the organ shortage, and related donation policies need to be standardised (Duvoux, 2019; O'Grady, 2018). Therefore, a large part of attention turns to animals, which can ensure sufficient, suitable size and high-quality organs. Although the application of gene editing and immunosuppressants have solved the problem of acute transplant rejection, the recipient of the first porcine heart transplant still regretted to die, which might be related to cytomegalovirus contamination of the graft (Mohiuddin et al., 2023). Avoiding virus infection, endothelial damage and antigen presentation are also challenges to be further explored in xenotransplantation.

The field of bioengineering organ tissue is still in its infancy, with much to be discovered about the complex structure and function of organs. The technical process must also be optimized in order to reduce the cost and time of manufacturing. Since the concept of tissue engineer or bioengineer was put forward in the 1990s, Joseph P. Vacanti had put forward three ideas about tissue engineering technology, which carry out engineering transformation at the cytokine level, the cell level and the tissue level (Langer and Vacanti, 1993). Based on these three level, some sophisticated and complex technics have been developed, including decellularization technique, 3D bio-printing, cell-sheet technique. Compared with the latter two techniques, decellularized scaffolds do not need to reconstruct the complex 3D structure of organs, and can retain microenvironment. From the successful decellularization of porcine liver reported in 2004 (Lin et al., 2004) to the decellularization of whole human liver (Mazza et al., 2015), decellularization has gradually become repeatable and mature. With this mind, Tissue engineering vascular grafts (TEVGs) can also be developed with decellularization and recellularization technologies. Though the commercial TEVGs products is available in hemodialysis and peripheral vascular replacement (Wang et al., 2022), the primary patency rate of vascular substitute is only 28% at 12 months (Lawson et al., 2016).

By searching and combining the medical subject terms “vessels,” “vascular” and “decellularization” in PubMed, we reviewed relevant experimental and clinical research articles since 2017. Meanwhile, the clinical use of decellularized vascular scaffolds was reviewed through ClinicalTrials.gov and the WHO’s International Clinical Trials Registry Platform (ICTRP), based on the recommendations made by Hunter et al. (Hunter et al., 2022) In this article, we focus on the decellularization treatments and their combinations of small blood vessels to review and discuss the impact of various vivo/vitro experimental data on extracellular matrix (ECM). Next we will summarize and discuss post-decellularization operations to optimize the performance of TEVGs *in vivo*/vitro.

## 2 Decellularization of vessel

### 2.1 Introduction

The goal of vessel decellularization is to remove all immunogenic cells from vessels of human (allogeneic) or other species (xenogeneic) and minimize the damage to the primitive ECM as much as possible. Scaffold materials provide a three-dimensional environment for tissue repairing and growing. The final purpose of the decellularized vessel is to assist the body to complete the structural remodeling and functionalization of the target vessels with a favorable immune response.

According to the decellularization materials, the decellularization techniques can be divided into two types: 1) Natural biomaterials; 2) Artificial materials. Most of the experiments reviewed in this paper are derived from natural tissue. But Jeffrey H Lawson and his colleagues had applied decellularized vessel proliferated and incubated from human-donor vascular smooth muscle cells (SMCs) to 60 end-stage renal patients during dialysis. (Lawson et al., 2016). This type of human-cell derived vessel is obtained by seeding donor expanded SMCs on biodegradable scaffolds and then undergoing decellularization treatment. From a certain point of view, it is more like the hybrid product of artificial vascular technology and decellularization technology, and it is indeed the most successful small vessel substitute at present in clinic. It had been used to treat peripheral vascular diseases and injuries (Lauria et al., 2022), which would have a far-reaching impact on the related fields of vascular surgery, transplantation, orthopedics and so on.

### 2.2 How to decellularize?

Since the main purposes of constructing decellularized vascular scaffolds is for subsequent recellularization, we need to pay attention to the following problems in the decellularization process. Firstly, decellularized xenogeneic tissues vary greatly in the degree of host response, mainly because of previous tissue processing steps, rather than because of their xenogeneic nature (Brown et al., 2012). Secondly, the main substances in ECM (such as fibronectin, collagen and laminin) are beneficial to the adhesion, differentiation and dispersion of various functional cells during recellularization, which is associated with growth factors, proteins, carbohydrates, as well as glycosaminoglycans (GAG) (Caires-Júnior et al., 2021). Therefore, maximum retention of ECM is another problem that needs to be focused on. The basic principle of decellularization is to separate the connection between the cell and the matrix and split the cell membrane so that the cell debris can be eluted from the 3D reticular structure. Therefore, we do not evaluate and summarize the advantages and disadvantages of single treatment methods again. (Dai et al., 2022). Combinations of different methods, detergents and enzymes tend to complement each other’s deficiencies and capitalize on each other’s strengths. Therefore, according to different combinations, we classify and discuss the decellularization treatments.

#### 2.2.1 Physics-enzyme-detergent combination

**FIGURE 1:**
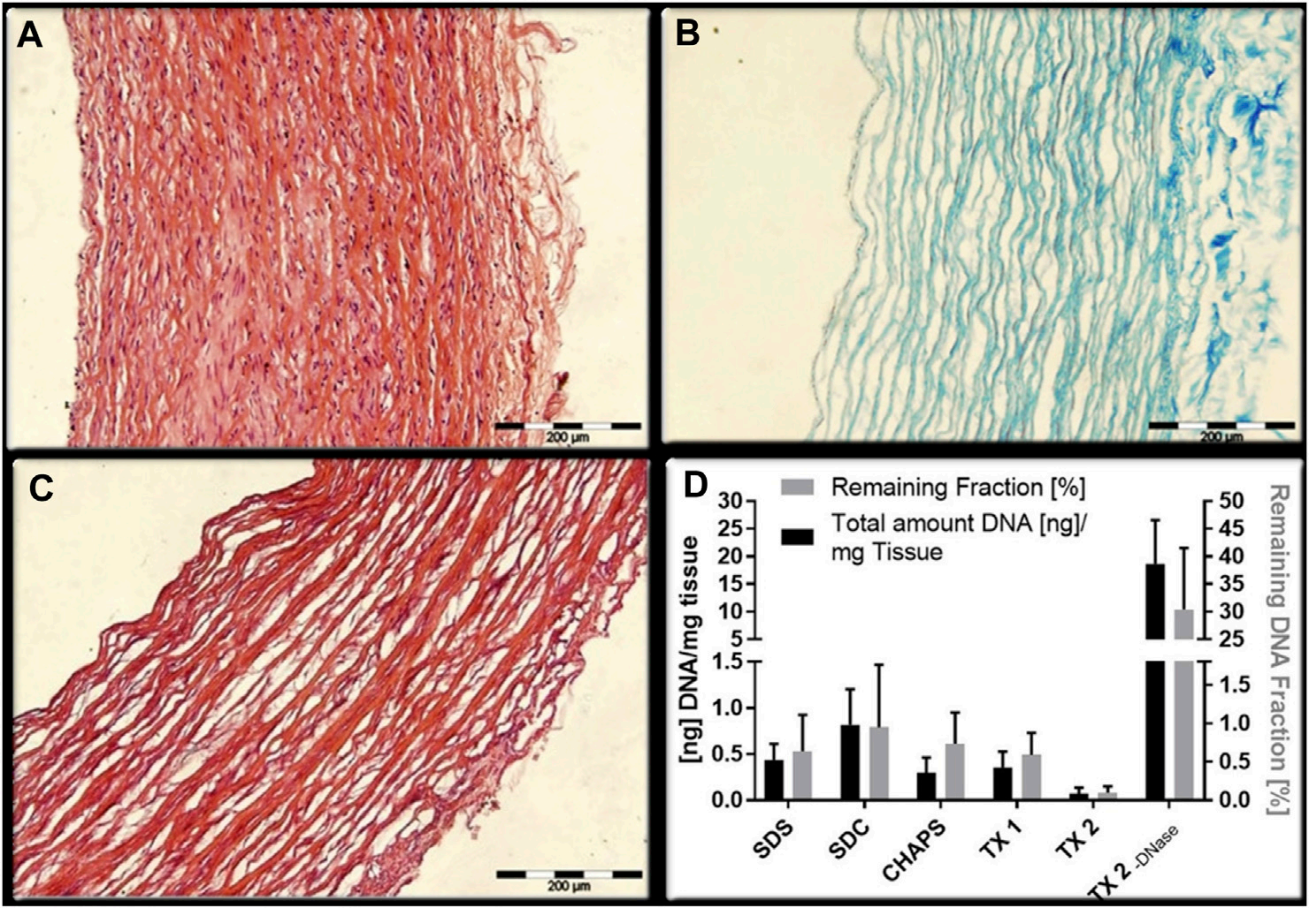
Comparison of two decellularization combinations. Although the decellularity were measured by microscope images analysis (López-Ruiz et al., 2017) **(A)**: HE staining of natural arterial blood vessel; **(B)** staining of collagen fibers remaining after decellularization; **(C)** HE staining after decellularization) or DNA quantification (Dahan et al., 2017) **(D)**: quantification of DNA and proportion of retained fraction after decellularization), they can both reflect the decellularity of the scaffold and the integrity of remaining ECM to a certain extent.

The physics-enzyme-detergent combination method is the most frequently used in most TEVGs experiments. And it can be observed that the treatment time of decellularization increases with the increase of the diameter and inner wall thickness of blood vessels. The inner diameter and inner wall thickness of blood vascular at the same site increased gradually from mouse to pig to sheep, and the arterial wall was thicker than the venous wall. Generally, the gross decellularization time is proportional to the cross-sectional area of the blood vessel wall and also depends on the formula of decellularization solution. When focusing on the decellularization of porcine carotid arteries (Table 1), the treatment time of López-Ruiz’s method is shorter and more efficient with the evaluation of histological analysis. (Dahan et al., 2017; López-Ruiz et al., 2017). In contrast (Figure 1), firstly, the method of removing cells by osmotic pressure is time-consuming. Secondly, properly increasing the concentration of TritonX-100 can also shorten the treatment time. However, the concentration and treatment time of trypsin related to the destruction of collagen, elastin and GAGs are observed (Lin et al., 2018), which has also been confirmed that even very low concentration of trypsin was also responsible for vascular histological changes. (Wang et al., 2021). Therefore, trypsin-hydrolysis may be not suitable for small or thin blood vessels, such as the vessel of Sprague-Dawley (SD) rat or mice. As for the physical methods, agitation or perfusion are efficient in facilitating contact of enzymes and detergents with tissues, even for sub-millimeter vessels such as the carotid artery of SD rats (Wang et al., 2021) and the human placental artery. (Falkner et al., 2023). When coming to evaluate the decellularization efficiency, histological analysis or DNA quantification were adopted. Although histological analysis can reflect the macroscopic structure of the tissue and evaluate the decellularization efficiency to some extent, it cannot be quantified and cannot reflect the overall situation due to the sampling deviation and heterogeneity of blood vessels in different parts.

**TABLE 1:** Summary of combinations of decellularization methods.

Donor source	Decellularization treatment	References
Porcine carotid artery	Physics-Enzyme-Detergent (agitation + hypertonic-hypotonic treatment + 0.05%Trypsin + 1% TritonX-100 + 1%NH_4_OH) for 270+hours	Dahan et al. (2017)
Porcine carotid artery	Physics-Enzyme-Detergent (agitation + 0.05%Trypsin +2% TritonX-100 + 0.8%NH4OH) for 73 h	López-Ruiz et al. (2017)
Carotid artery of rabbit	Physics-Enzyme-Detergent (freezing-Thawing + 0.125%Pepsin + DNase-RNase +1% TritonX-100) for 105–107 h	Xu et al. (2017)
Carotid artery of rat	Physics-Enzyme-Detergent (agitation + freezing-thawing + 0.125%Trypsin + 70%ethanol) for 55.5 h	Wang et al. (2021)
Vena cava of pig	Physics-Enzyme-Detergent (agitation + 1%TritonX-100 + 1%Tri-n-butylphosphate/TNBP + DNase) for 240 h	Håkansson et al. (2021)
Carotid artery of sheep	Physics-Enzyme-Detergent (agitation + TritonX-100 + Tri-n-butyl phosphate/TNBP + DNase) for 9 days	Jenndahl et al. (2022)
Coronary artery of ovine	Physics-Enzyme-Detergent (agitation + hyperosmotic + 1%TritonX-100 + 0.025%Trypsin) for 220 h	Omid et al. (2023)
Bovine internal mammary artery	Physics-Enzyme-Detergent (perfusion + sonication + 0.5%SDS + 0.5%TritonX-100 + 40U/ml DNase + 0.3 mg/ml RNase) for 108.5 h	Liu et al. (2022)
Human placental artery (<1 mm)	Physics-Enzyme-Detergent (perfusion + hyperosmotic treatment + 1%TritonX-100 +DNase) for 30 h	Falkner et al. (2023)
Arterial branches of rabbits	Enzyme-Detergent (0.1%Trypsin +1% TritonX-100) treatment for 78 h	Liu et al. (2017)
Abdominal aortas of rabbits	Enzyme-Detergent (0.1%Trypsin + 1%TritonX-100 + 20 μg/mL RNase + 0.2 mg/ml DNase) for 75–77 h	Kong et al. (2019)
Porcine carotid arteries	Physics-Enzyme (agitation + 0.5%Trypsin) treatment for 48 h	Gu et al. (2018)
Coronary arteries of pigs	Physics-Detergent (agitation + 1%SDS + 1%TritonX-100) for 120–168 h	Du et al. (2022b)
Carotid arteries of pigs	Physics-Detergent (freezing-thawing + agitation+ 1%Triton-X 100 + 0.3%SDS) for 84+ hours	Cheng et al. (2023)
Human umbilical cord artery	Physics-Detergent (sonication + 2%SDS for 4 h) for 49 h	Lin et al. (2021)
Common carotid arteries of rats	Enzyme (0.15%Trypsin + DNase + RNase + lipase) treatment	Huo et al. (2017)
Common carotid arteries of rats	Enzyme (0.15%Trypsin + DNase + RNase + lipase) treatment	Chen et al. (2018)
Human umbilical cord artery	Detergent (2%TritonX-100 + 0.1%SDS) treatment for 72 h	Muniswami et al. (2020)
Porcine aortic wall tissue	Detergent (1%SDS) for 48+ hours	Lehmann et al. (2017)

#### 2.2.2 Enzyme-detergent combination

In the Enzyme-Detergent binary-decellularization, the combination of trypsin and TritonX-100 is applied coincidentally. The effect of trypsin on the ECM of porcine carotid artery was less than that of SD rats, and no significant change in the arrangement of collagen fibers was observed (Liu et al., 2017), which is shown in Table 1. However, neither TritonX-100 nor trypsin can remove nucleic acid very well. In the experiments of Kong et al., 2019, with the addition of DNase and RNase, the immune response induced by scaffolds was greatly reduced to guarantee the continuous patency of blood vessels. But the mechanical property results indicated the possible loss of ECM components and structural damage, which might due to the application of trypsin.

#### 2.2.3 Physics-enzyme combination

The experiment used a single-enzyme to decellularize combined with physical methods to speed up the process. In the treatment of porcine carotid arteries, compared with the physics-enzyme-detergent method or the enzyme-detergent method, the concentration of trypsin needed for decellularization is higher. The analysis of histological and mechanical properties was applied to evaluate the experimental results. The results showed that the decellularization was not complete and was accompanied by the destruction of collagen fibers. not only the strength was insufficient, but also the exposed collagen fibers were easy to cause immuno-inflammatory response of the receptor.

#### 2.2.4 Physics-detergent combination

For porcine arteries, the combination of chemical and physical methods in Table 1 not only removed the cells and residual DNA, but also maintained the integrity of collagen, GAGs and their microstructure. The results of cytotoxicity and biomechanical test also suggested that although there were changes in TEVGs, they were not statistically significant from natural blood vessels. It was proved that for the vascular tissue with the thickness of porcine coronary artery or carotid artery, the addition of SDS can make up for the incomplete decellularization defect of TritonX-100, and the damage of SDS to ECM can be alleviated to the minimum by controlling the treatment time and concentration. Notably, in this section, the application of ultrasound took decellularization efficiency to a new level. With controlled power and time, the same thickness of tissue could be decellularized in a very short period of time with complete retention of the ECM. (Lin et al., 2021). However, the disadvantage was that it took several times longer than decellularization processing to clean up the remaining cellular debris.

#### 2.2.5 Enzyme-only

These two studies included in this group adopted the same mild protocol (Table 1) in the selection of decellularization enzymes. (Zeng et al., 2012). Although the experiments were lack of evaluation of graft decellularization efficiency and cytotoxicity analysis, but in the follow-up experiments, the graft could maintain a medium and long term patency rate of 80%–90% in the *in vivo* transplantation experiment with different surface modification.

#### 2.2.6 Detergent-only

Histological evidence to prove no nuclear residue was performed in this experiment, and DNA quantitative analysis was also established, suggesting reasonable detergents treatment could achieve considerable decellularization efficiency. Especially, RNA quantitative was mentioned for the first time in the article for evaluation. (Lehmann et al., 2017). Compared with DNA, since RNA exists widely in nucleus, cytoplasm and even extracellular, it may be more representative of the thoroughness of decellularization.

#### 2.2.7 Non-mainstream method

In addition to the conventional decellularization methods and their combinations mentioned above, innovative decellularization methods are constantly being tried. In the supercritical state, carbon dioxide exists as a fluid with gas-liquid characteristics. In this state, supercritical carbon dioxide can take away the lipids in the femoral artery of rabbits in 90 min (chemical treatment of the same tissue requires 72 h and 6 days of washing) to achieve decellularization (Sung et al., 2023). The results of *in vivo* experiments also verified that supercritical fluids retained the intact mesh structure of ECM compared to SDS. The same principle was applied to subcritical dimethyl ether to extract lipids from tissues, but data from *in vivo* experiments are not available (Kanda et al., 2021).

In recent years, an alternative method has been proposed in which tissues were decellularized by inducing apoptosis. And in the related experiments of decellularized nerve and lung tissue, camptothecin is used to induce cell lysis into small apoptotic bodies to make it easier to remove cell fragments and maximize the preservation of extracellular matrix without detergents (Cornelison et al., 2018; Song et al., 2021). However, if the apoptotic products which is usually engulfed by macrophages *in vivo* are not cleaned quickly, it will lead to secondary necrosis (Nagata, 2018), which will cause damage to the ECM and make the whole decellularization non-efficient. However, as a potential decellularization methods of vascular, the data in vessel is limited.

#### 2.2.8 Summary

The truth is complete removing the cellular components from the tissue is an almost impossible feat, so the realistic approach is to find a suitable treatment according to the characteristics of the tissue, so as to achieve a delicate balance between decellularization and structure protection. From the included studies, the use of multiple decellularization methods in combination with each other depending on the thickness of the vessel has been adopted by many research teams (Figure 3). The combination of multiple methods can alleviate the side effects of a single method by reducing the time or concentration of single method treatment to main the balance properly.

In the physical method, static, perfusion and agitation are the common methods used for decellularization. For vascular decellularization, decellularization by perfusion can quickly remove degraded cells to improve efficiency, but it is not “the faster, the better”. It had been studied that vascular endothelium was easy to be torn at high perfusion rate, which would lead to thrombosis after transplantation, and there seemed no significate difference in decellularization of large diameter blood vessels by the above mentioned ways. (Simsa et al., 2019). But according to Poiseuille’s law, this is not the same condition in small diameter vessels. In blood vessel models with larger diameters, such as porcine vena cavas (inner diameter: 13.11 mm ± 0.96 (Simsa et al., 2019)), the detergents can flow into the blood vessels with very little pressure in the decellularization process, whether by immersion, vibration or perfusion. In small-diameter blood vessels (porcine carotid arteries: 3–4 mm; carotid arteries of rabbits: 1.8–2.2 mm; mouse carotid artery: 0.6–0.9 mm), although the inner diameter is only reduced by a few millimeters, the changes are super multiplied, which results in insufficient fluid spreading into the blood vessels to take away the cell fragments and leads to inefficiency. Notably, in organs with complex vascular networks, such as the liver, the decellularization efficiency is not only related to the concentration of the solution and the time, but also observed that the perfusion method is more efficient, and different input channels have different decellularization efficiency. (Struecker et al., 2017; Panahi et al., 2022). Among physical methods, ultrasound has demonstrated unparalleled efficiency and robust ECM protection in the field of decellularization in recent years, making it a reliable and rapid method.

Among the reported decellularization detergents, although most experiments used ionic detergent is sodium dodecyl sulfate (SDS), non-ionic detergent, such as Triton X-100, showed better tissue protection and lower cytotoxicity. Scaffolds treated by SDS contained less dsDNA than other detergents, which was related to its strong decellularization ability and protein denaturation properties (White et al., 2017). Triton X-100 disrupts DNA-protein, lipid-lipid and lipid-protein interactions and protect protein activity, especially for the preservation of basement membrane and fiber network integrity, which would facilitate cell adhesion and accelerate the progression of recellularization (Faulk et al., 2014; Simsa et al., 2018). Meanwhile application of DNase was suggested after TritonX-100 treatment for thorough DNA removal (White et al., 2017; Simsa et al., 2018). Zwitterionic detergents mentioned in the article are 3-[(3-cholamidopropyl) dimethylammonio]-1-propane sulfonate (CHAPS) and tri-n-butylphosphate (TNBP). But when compared with TritonX-100, zwitterionic detergents are not effective enough in e porcine vena cava decellularization (Simsa et al., 2018). Generally speaking, the longer the contact time between the detergents and the tissue under same physical environment, the deeper the degree of infiltration with the better decellularization efficiency (Crapo et al., 2011; Cheng et al., 2021).

Enzymatic hydrolysis is also an effective decellularization method with strong characteristics and low cytotoxicity. However, as mentioned above, the wrong thickness of blood vessels matched with the wrong concentration and processing time can cause damage to ECM, although the damage is partially reversible. So, it is suggested that the mechanical properties analysis, including longitudinal tension, burst pressure, suture tensile strength and so on, is necessary after trypsin-containing treatment to evaluate the overall structural strength of materials. The application of nuclease is also a controversial issue. After decellularized vessel transplantation, released extracellular nucleotides may trigger inflammation and lead to an inflammatory microenvironment. So from this point of view, it is necessary to clean the nucleotides. After nuclease treatment, matrix proteins were found to be seriously lost, which indicates the activation of proteases during nuclease incubation (Mangold et al., 2015). Similarly, Simsa, R. and his colleagues found that the use of DNase was related to the decrease of mechanical stability and the decrease of GAGs content (Simsa et al., 2018). But the deletion of nucleotidase is suspected to be a potential factor for insufficient of decellularization, and a large amount of nucleic acid material would still be retained in the tissue, which would inevitably lead to immune rejection. Nevertheless, Ji Bao et al., 2015 had claimed the nucleotidase-free decellularization protocol was proved to be effective in perfusion decellularization of whole liver scaffolds, the residual DNA content was controlled at 30 ± 10 ng/mg and GAGs was well preserved.

**FIGURE 2:**
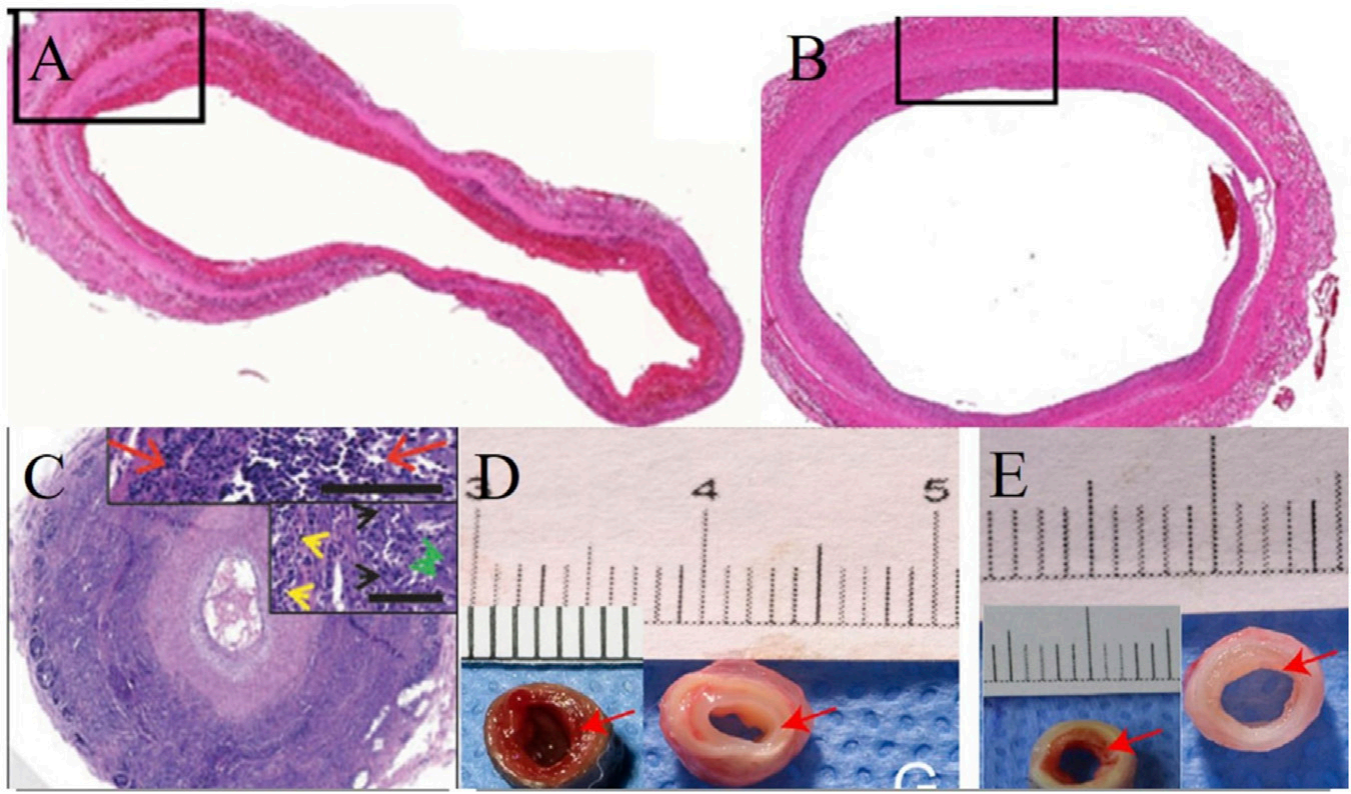
Effect of different treatments and time on remodeling of decellularized blood vessels *in vivo*. **(A,B)** Following 49 h of decellularization of human umbilical cord arteries, the internal diameter of the vessels remained almost unchanged and patent on days 3 and 90 *in vivo*. (Lin et al., 2021); **(C)** After 270 h of decellularization, the porcine carotid artery was almost occluded at week six *in vivo* (Dahan et al., 2017); **(D,E)** After 108.5 h of decellularization, bovine mammary arteries implanted *in vivo* for 4 weeks showed different degrees of endothelial proliferation before and after post-decellularization treatment (Liu et al., 2022).

In conclusion, the effect of decellularization treatment on blood vessels directly affects their remodeling *in vivo*. By comprehensively analyzing and comparing the pathological results of blood vessels from different tissue sources under different decellularization treatments, we found that minimizing the exposure time of vascular tissues to decellularization improves their biocompatibility and at the same time improves their performance in the animal circulatory system (Figure 2).

When it comes to the storage of unprocessed or processed vessels, long storage (48–72 h) at 4°C would promotes continuous metabolism of cells and induces cell death or apoptosis which changed the PH value of the microenvironment in the organization and reduced the biocompatibility of the scaffolds, but fetal bovine serum continuous flushing would reverse this effect. (Omid et al., 2023). However, the increase of cytotoxicity did not occur at-20°C storage temperature. On the contrary, after clean and sterilization, the storage at 4°C did not show different results in the cytotoxicity test, and it also performed well in the *in vivo* transplantation experiment (Liu et al., 2022).

**FIGURE 3:**
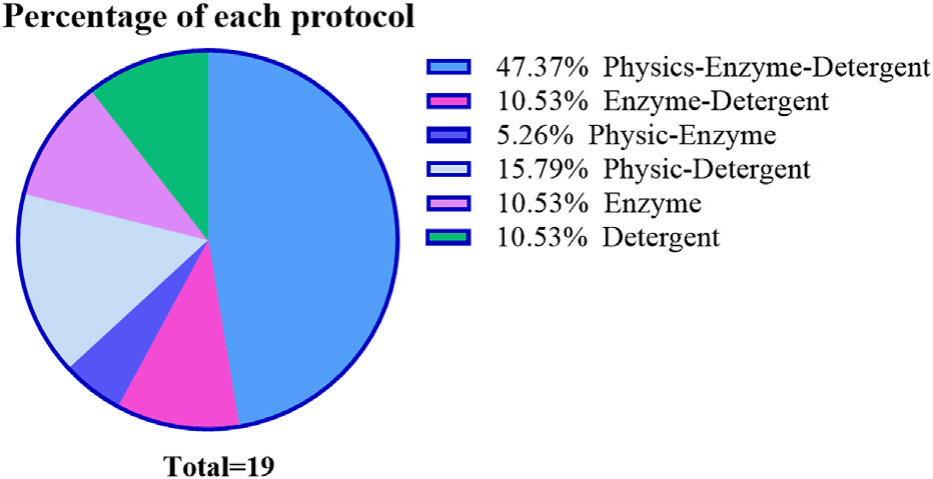
Proportion of different decellularization methods based on included experiments.

When we discuss with the evaluation of decellularization efficiency, there is no unified method. The conventional evaluation methods are mainly microscope image analysis and DNA quantitative (Figure 1). A residual DNA level less than 50 ng dsDNA per mg of dry weight of the ECM scaffold, which is generally considered to be the standard for successful decellularization in different organ. However, the antigenicity of the scaffold cannot be fully evaluated by assessment of residual cellularity (via residual nuclei counts under light or fluorescence microscopy) or residual DNA quantification in the tissue. Related studies of heart valves have suggested that MHC-I and galactosea (1,3)-galactose (α-gal) antigens can still be detected after decellularization, which bound to chronic graft rejection (Goldstein et al., 2000). And there was evidences that even if reaching the decellularization standard at DNA level, enzyme-linked immunosorbent assay result still showed a similar titer in undecellularized group (Park et al., 2013). Nevertheless, the outcomes of all *in vitro* tests must be validated by *in vivo* experiments. As illustrated in Figure 3, although the scaffolds were successfully decellularized at both the quantitative and qualitative levels, the degree of endothelial hyperplasia within the circulatory system exhibited considerable variability.

### 2.3 What are the obstacles we face?

The ideal TEVGs have well-established low immunogenicity, surface physical and mechanical properties, meanwhile appropriate biodegradability and biomechanical properties are indispensable. But the reality is counterproductive.

Like prosthetic vascular grafts, coagulation causes the main graft failure at early stage, while Intimal hyperplasia is the main cause in the later graft failure. (Zilla et al., 2007). Intimal hyperplasia always occurs on the back of existing tissue, which is the anastomosis in prosthetic vascular grafts. But in the TEVGs, because the whole graft has the potential of regeneration and remodeling, so intimal hyperplasia can occur any segment in the whole vessel. Except for the activation of chronic inflammation (Chen et al., 2018), due to the loss of subtle proteins, the mechanical properties of decellularized vascular scaffolds will also undergo subtle changes. (Omid et al., 2023). Mechanical changes (Cheng et al., 2021) and cross-sectional quotient (Qc) mismatch (Zilla et al., 2007) eventually lead to intimal hyperplasia, which also occurs when there is no evidence of immune rejection. (Jenndahl et al., 2022).

Overcoming immunogenicity was the original intention of establishing decellularization technology, but contrary to our wishes, the immune response wound still take place stubbornly in the decellularized scaffold, like a ghost. On the one hand, the persistence of the MHC I and α-gal antigen in the decellularized scaffold indicates that the remaining cell debris still exist in the scaffold, demonstrating not only the deficiency of decellularity as a sole outcome measurement (Wong and Griffiths, 2014), but also the inadequacy of current decellularization regimens. On the other hand, the α-gal that naturally exists in the matrix makes it unrealistic to completely remove all the immunogenicity (Stahl et al., 2018).

*In vivo* calcification is another main cause of graft failure. The exact mechanism is unclear and involves a number of factors that can be broadly categorized as decellularization, post-decellularization and receptor related factors. It is well-known that decellularization is destructive to the ECM. Exposed collagen and elastic fibers in TEVGs adsorb calcium ions *in vivo*, resulted in calcium deposition (Pai et al., 2011). Therefore, prior to implantation in the body, it is necessary to subject the material to post-decellularization treatment. However, it is also important to select appropriate modifications. For instance, the cross-linking agent glutaraldehyde has the potential to accelerate the calcification of the implant (Meuris et al., 2003). It is noteworthy that not only do implant-related factors accelerate calcification, but physiological factors of recipient also react chronically with the scaffolds. Abnormal glycosylation end products caused by diabetes can cross-link with collagen and elastic fibres in blood vessels, which not only increases the stiffness but also accelerates the calcification of scaffolds (Chow et al., 2013).

In addition to immune rejection and calcification leading to degradation, after decellularization, the fracture of the original cell-collagen junction not only leads to changes in mechanical properties, but also may make decellularization blood vessels more easily degraded in vessels. During vascular remodeling, the infiltration of macrophages and enzymatic degradation of the blood vessel wall would result in further cracking (Lehmann et al., 2017). The time of degradation is shorter than that of remodeling would lead the hemangioma in TEVGs (Cornelison et al., 2018). Under the influence of arterial pressure, any decrease in mechanical properties or degradation of ECM over synthesis will lead to dilatation of the blood vessel wall and eventually lead to failure.

For the natural blood vessels, a functional and intact endothelial layer can antagonize the formation of thrombosis by synthesizing and secreting prostacyclin (PGI2), endothelin, etc. Non-endothelialized vascular scaffolds could be found to have extensive clotting and adverse remodeling 6 weeks later after transplantation. But after endothelialization, the recovery of mechanical properties could be observed, which might due to the major contribution of SMCs from recruited host cells (Dahan et al., 2017). But endothelialization is a relatively slow process which cannot be functional after seeding immediately and requires long-term *in vitro* culture.

### 2.4 How to overcome?

In the process of decellularization, the inability to monitor the decellularization process in real time results in the unavoidable negative effects on ECM. Although a small percentage of unmodified vessels have been experimentally shown to maintain long-term patency *in vivo*, post-decellularization treatment is necessary prior to clinical application. In this section, according to the process, the methods are divided into three groups: *in vitro* recellularization, surface modification and a combination of the two.

#### 2.4.1 Recellularization *in vitro*

The loss of endothelial cells (ECs) and smooth muscle cells (SMCs) of decellularized scaffold causing collagen exposure in bloodstream after transplantation, which inevitably leads to thrombosis and low patency, is still a major problem in the application of vascular transplantation (Dahan et al., 2017; Hsia et al., 2017). Given the essential role of endothelium in preventing vascular occlusion, it is understandable that the main focus of vascular transplantation research is endothelialization. Firstly, it is important to note that vascular ECs are heterogeneous among different organs. For example, liver ECs (also known as hepatic sinusoids) have fenestrae structure, which are associated with hepatocyte function and liver diseases (Braet and Wisse, 2002). Re-endothelialization of vascular ECs with special structure and function, such as in the liver or kidney, is very different from normal vascular endothelialization and much extremely challenging. We therefore focus on the re-endothelialization of the normal vascular. In fact, endothelialization has two ways: 1) to induce the migration and germination of neighboring ECs; 2) to recruit endothelial progenitor cells (EPCs) from circulation. Moreover, in view of the fact that the endothelial growth across the anastomosis is less than 1–2 cm in the humans (Zilla et al., 2007), the latter mechanism plays a dominant role in the early endothelialization.

In the strategy of vessel recellularization, most experiments are realized by seeding exogenous autologous (Dahan et al., 2017), allogeneic or xenogeneic cells (López-Ruiz et al., 2017; Muniswami et al., 2020; Omid et al., 2023). For scaffolds without pretreatment *ex vivo*, endothelialization *in vivo* takes weeks or longer, although patency can be maintained for a certain period of time (up to 90 days) (Martin et al., 2005; Lin et al., 2021). However, endothelial hyperplasia and fibrosis might have an effect on the patency of the grafts. *In vitro* recellularization helps the grafts maintain long-term patency to meet clinical needs. For the selection, numerous cell lines can be chosen and co-cultured in the scaffolds, e.g., vascular endothelial cells and smooth muscle cells, endothelial progenitor cells. Although simulating the intravascular physical environment and providing hypocoagulable environment *in vitro* could accelerate cell infiltration and shorten the culture cycle, the time might still be more than 3 weeks (Table 2). Accelerating the adhesion of ECs is one solution.

**TABLE 2:** Summary of endothelialization methods.

Donor source	Post-decellularization treatment	Patency period	References
Porcine carotid arteries	Autologous venous endothelial cells (VECs) and arterial smooth muscle cells (ASMCs) cells were seeded into lumen and tunica externa respectively for 21days	Up to 6 weeks (Allotransplantation)	Dahan et al. (2017)
Porcine carotid arteries	After treatment with ethylmethacrylate-co-diethylaminoethyl acrylate coating, human umbilical vein endothelial cells (HUVECs) were seeded and and incubated for 5 days	N/A	López-Ruiz et al. (2017)
Human umbilical cord artery	Human umbilical cord endothelial progenitor cells (HUCEPCs) and smooth muscle cells (HUCVSMCs) were seeded into lumen and dorsal surface respectively for 24days	At least 7 days before execution (Xenotransplantation)	Muniswami et al. (2020)
Coronary arteries of ovine	After rat thoracic aortic smooth muscle cells (A10 cell line) were implanted, they were cultured in a bioreactor to simulate the physical environment of blood vessels *in vivo*	N/A	Omid et al. (2023)

CD34^+^CD113^+^ Endothelial progenitor cells (EPCs) with the potential to proliferate and differentiate into ECs can be used to reconstruction of endothelial integrity (Siavashi et al., 2017). It had been proved that hematopoietic stem and progenitor cells express markers similar to EPCs, such as CD133, CD34 and so on (Peichev et al., 2000; Friedrich et al., 2006). Based on this, the anti-CD34 antibody coated vascular graft can recruit circulating endothelial progenitor cells to accelerate endothelial repair and reduce thrombosis (Chen et al., 2012). Similarly, applying vascular grafts modified with anti-CD133 antibodies which present higher ability of proliferation in CD34 subset (Bachelier et al., 2020) can also accelerate EPCs attachment (Lu et al., 2013). Except for the antibody method, stimulating adhesion signaling pathway is also a solution. Sphingosine-1-phosphate modification accelerate EC adhesion by activating MMP2/FGF-1/FGFR-1 pathway (Hsia et al., 2017). In addition to combining with natural materials, decellularized scaffolds coated with ethylmethacrylate-codiethylaminoethyl acrylate show gratifying results when comparing with control group in EPC adhesion and Platelet adhesion (López-Ruiz et al., 2017).

#### 2.4.2 Surface modification

In the exploration of endothelialization, whether the functional cells that make up the surface of TEVGs are original seeding cells? In contrast to the prevailing view, evidence indicated that endothelial functionalization was not contingent on the presence of implanted cells, but rather on inflammatory remodeling cells recruited by cytokines (Roh et al., 2010). The implanted cells more likely acted as a convener to recruit repair cells of receptor. From the point of view of time-consumption and reliability, surface modification can target at different defects such as inflammation, thrombosis, immunogenicity, mechanical properties and cell adhesion to enhance TEVGs performance by crosslinking or coating (Table 3) natural, artificial or chemical materials.

**TABLE 3:** Post-decellularization methods including cross-linking, coating and pre-condition.

Donor source	Post-decellularization treatment	Patency period	References
Common carotid arteries of rats	Modification of collagen-coated reduced graphene oxide based dual-enzyme system	90% (7-days patency rate) (Allotransplantation)	Huo et al. (2017)
Arterial branches of rabbits	Combining with 0.25%human-like collagen I by freeze-drying after photooxidant cross-linking	N/A	Liu et al. (2017)
Carotid arteries of rabbits	UV irradiation	N/A	Xu et al. (2017)
Common carotid arteries of rats	Combination collagen-coated vessel with EDC and CGS21680	80% (6-month patency rate) (Allotransplantation)	Chen et al. (2018)
Porcine carotid arteries	Cross-linking with genipin (0.2% for immersing 6 h)	N/A	Gu et al. (2018)
Abdominal aortas of rabbits	Heparin immobilization with EDC and NHS and incubating with VEGF and bFGF.	90% (18-month patency rate) (Xenotransplantation)	Kong et al. (2019)
Carotid arteries of rats	Photooxidant cross-linking with methylene blue and hydrogen peroxide	N/A	Wang et al. (2021)
Carotid arteries of pigs	Binding to HGF after surface heparinization with EDC and NHS crosslinked	N/A	Cheng et al. (2023)
Porcine aortic wall tissue	Binding to FGF or VEGF after surface heparinization with EDC and NHS crosslinked	N/A	Lehmann et al. (2017)
Bovine internal mammary artery	Photooxidation and pentagalloyl glucose (PGG) cross-Linking	100% after 4weeks (Xenotransplantation)	Liu et al. (2022)
Coronary arteries of pigs	Hydrogel was used to coat the dorsal surface and cross	N/A	Du et al. (2022b)
Vena cava of pigs	Perfusion of recipient autologous blood, organ preservation solution and cytokine mixture for 7 days	Up to 5 weeks (Allotransplantation)	Håkansson et al. (2021)
Carotid arteries of sheeps		Up to 4 months (Allotransplantation)	Jenndahl et al. (2022)

##### 2.4.2.1 Anti-inflammation-based modification

Regulation of host inflammatory response to grafts helps to maintain long-lasting patency of vascular. The mononuclear macrophages recruitment induced by CCL2 in the early stage and vascular remodeling induced by mononuclear macrophages in the later stage of vascular graft maturation help us to comprehend the significance of inflammation (Roh et al., 2010). Adenosine A2a receptors regulate arterial remodeling by maintaining recruited macrophages retention (He et al., 2020), which can be performed by adenosine receptor agonists CGS21680 to inhibit matrix-induced inflammation (Scheibner et al., 2009). Tissue engineered blood vessels achieving slowly releasing CGS21680 by cross-linking with drug-load nanoparticle had confirmed the increase of M2-type (anti-inflammation type) macrophage percentage.

##### 2.4.2.2 Anticoagulant-based modification

ADP can be released immediately by activated platelets (von Papen et al., 2013). As a key link of platelet activation, ADP can enhance other pathways of blood coagulation. AMP and adenosine can antagonize or even reverse platelet aggregation induced by ADP (Born, 1962). So from this point of view, transferring ADP into AMP or adenosine can become an antiplatelet target for surface modification. This hypothesis has been verified in an animal model. Through the transformation of ADP into AMP and adenosine by double-enzyme system, there was almost no platelet aggregation or thrombosis on the endothelial surface modified by reduced graphene oxide after 7 days *in vivo*. (Huo et al., 2017). At the same time, although not mentioned in the article, the transformed adenosine can mediate the anti-inflammatory system of macrophages by activating adenosine A2a receptor, which promotes the graft remodeling *in vivo*. (Scheibner et al., 2009).

Heparin is widely used in anticoagulation therapy in clinic. Heparin immobilization had also been shown to optimize the antithrombotic ability of intrahepatic vessels in scaffolds. (Bao et al., 2015). However, the heparin immobilization will always be exhausted *in vivo* and cannot be compensated. In small diameter arteries, heparinization alone could not optimize the patency rate in the later stage (10% after 12 months), but the addition of VEGF and FGF significantly changed the patency rate of engineered vessels, reaching 90% patency after 18 months (Kong et al., 2019). Therefore, the release period of heparin must cover the remodeling period of TEVGs to ensure the patency of early stage.

##### 2.4.2.3 Lower-immunogenicity based modification

Although cells are removed from tissues after decellularization by evaluating of light or electron microscopy, and even the amount of residual nucleic acid was less than the standard of 50 ng/mg, the immune response is still observed which we have discussed above. The ideal solutions before us are as follows: 1) cross-linking; 2) enzymatic removal; 3) transgenic modification. Lack of α-gal epitopes in humans and primates leads to high levels of circulating anti-α-gal antibodies that specifically interact with heterogeneous α-gal epitopes to produce rapid, complement-driven hyperacute rejection of xenogeneic tissues. α-galactosidase removal of α-gal epitopes may provide a promising solution, but the experiments in other tissue demonstrated incompletely removal of α-gal in ECM after enzymatic clearance (Xu et al., 2009). Although no acute rejection and short-term graft failure was observed, it had the potential to induce the increase of antibody titer and cause chronic rejection. Therefore, it is further proposed that the decellularization treatment to organs of α-(1, 3)-galactosyltransferase knockout pigs would theoretically obtain the active tissue without immunogenicity, which turned out no significant advantages in immunity (Xu et al., 2009; Gasek et al., 2021). This also raises a question to be explored that whether it is necessary to use gene knockout pigs to prepare TEVGs?

As for cross-linking, it can reduce the immunogenicity of grafts mainly due to hindrance of cross-linked fibers (Luo et al., 2021; Cheng et al., 2023). By comparing the inflammatory response, cross-linked vascular scaffolds showed milder inflammatory cell infiltration and regular medial arrangement after 4 weeks compared with uncross-linked decellularized scaffolds. (Gu et al., 2018). As an earlier cross-linking agent, glutaraldehyde (GA) not only has high cytotoxicity, but also leads to immune rejection. (Chang et al., 2011). Therefore, apart from GA, ultraviolet (UV) cross-linking method can avoid the side effects of chemical cross-linking method. The UV irradiation can reverse the destructive effect of enzyme on ECM and maintain the mechanical properties of decellularized scaffolds. (Xu et al., 2017; Wang et al., 2021). But UV cross-linking could accelerate the degradation of vascular scaffold (Xu et al., 2017), which might due to the incomplete cross-linking of elastic fibers causing collapse of the structure of vessel wall in early stage. (Lü et al., 2010). And pentagalloyl glucose (PGG) which was considered to be compatible with elastic fibers could avoid such collapse. (Liu et al., 2022). Another agent extracted from natural plants, genipin, was verified to maintain the vessel stress-strain property (Gu et al., 2018) and to promote regeneration. (Du A. et al, 2022). The cross-linking of proteins using 1-ethyl-(3-dimethylaminopropyl) carbodiimide (EDC) and N-hydroxysuccinimide (NHS) has been demonstrated to reduce macrophage infiltration by forming barriers (Lehmann et al., 2017), while it also hindered the migration of repopulated cells and growth factors from receptor (Lü et al., 2010), which may delay the functionalization of vascular.

As an alternative, denudation of vascular endothelium can preserve the integrity and contractility of vascular wall while protecting vascular regeneration ability compared with enzyme treatment and hypotonic treatment (Hoenicka et al., 2013). The procedure has the potential to optimize the functionality of blood vessels. However, it is important to note that the risk of residual xenogeneic cells inducing hyperacute rejection is also a concern. At present, *in vivo* data is limited.

##### 2.4.2.4 Enhanced mechanical properties

Mismatching mechanical properties between TEVGs and native vessel can lead to graft failure acutely or chronically. UV irradiation, photooxidant/chemical crosslinking or coating, can improve the stiffness of TEVGs and reduce the compliance, so as to match to the natural blood vessels. Normally, TEVGs experiments’ mechanical testing include elastic modulus, suture strength, burst pressure, for vascular scaffolds, it is important to avoid degradation before restoring certain degree of function and structure. Consequently, while biocompatible cross-linking agents are employed to enhance mechanical performance, they also serve to delay the degradation of scaffolds *in vivo*. While the crosslinking or coating increased the extensibility and reduced the stiffness of the material, it was at the cost of reducing the porosity (especially for 10 μm in diameter) which served as channels for the infiltration of regenerated cells (Wang et al., 2021; Cheng et al., 2023). Therefore, although a unified evaluation to balance the mechanics and biocompatibility is absent, there is a principle that can be used for reference that sufficient porosity needs to be ensured after cross-linking or coating.

##### 2.4.2.5 Growth-factor based modification

ECM had been proven to induce stem cells to differentiate into ECs with cytokine supplements (Ullah et al., 2019). Combined with cross-linking and heparin immobilization (Kong et al., 2019), the patency rate after transplantation *in vivo* was much higher than that in endothelialization group. In addition to growth factor supplements, the preconditioning of scaffolds by perfusion of peripheral blood mixture from the receptor also achieved long-term patency for 4 months *in vivo* (Table 3). The principle was assumed to be the same as growth factors adhering to the surface of blood vessels, which was beneficial to its remodeling *in vivo* (Håkansson et al., 2021; Jenndahl et al., 2022). It is worth noting that the mixture of blood, organ-preservation solution and growth factors forms a bio-layer on the intima during several days of perfusion, hindering the direct contact between the recipient blood and exposed collagen (Österberg et al., 2023). This approach has been successfully used in animal experiments of pigs and sheep.

## 3 Conclusion

It has been estimated that approximately 20% or more of surgical bypass grafts lack adequate caliber autografts (Beckman et al., 2021). In the event of a shortage of autologous blood vessels, the other option available to the surgeon, in addition to the almost non-existent donor vessels, is the use of bioengineered blood vessels. The current failure of artificial vascular grafts in small-diameter vessels as well as in sub-millimeter microsurgery continues to stimulate the development of ideal alternatives in this field. Xenotransplantation technology is a breakthrough to solve the problem of organ shortage, but how to solve the problem of xenogeneic organ immune rejection is an important difficulty hindering the development of this technology. Decellularized vessel can greatly reduce the immunogenicity of xenogeneic organs and are emerging as a promising and reliable TEVGs. Unlike artificial tissue, decellularized vessels from natural tissue represent a compromise between *in vivo* biological activity and *in vitro* tissue engineering. After treatment of detergent, enzyme or physics *in vitro*, growth factors, such as fibroblast growth factor 2 and transforming growth factor β1, can still be preserved in the scaffolds (Conconi et al., 2004). As we mention above, different decellularization approaches may correspond to different compositional and original structural damage, which will influence the immunogenicity, mechanical, cytotoxicity, regeneration. The combination of methods in the decellularization process is the future of decellularization technology. Physical approaches can shorten the exposure time to detergents and enzymes to reduce cytotoxicity and improve biocompatibility. And *vice versa*, chemical and enzymatic treatments also shorten the time of physical approaches and minimize the damage to the 3D structure of ECM and promote the remodeling process *in vivo*.

In addition to immunogenicity, the mechanical characteristics of the substitute products of small vessels are also important evaluation criteria, and researchers had put forward some evaluation systems (Camasão and Mantovani, 2021). Notably, decellularization is only the first step in bioengineering organ technology. The preparation of scaffolds with low immunogenicity, low cytotoxicity and high biocompatibility cannot be directly applied in transplantation. The tentative recellularization of human-scale scaffold through the vessel network retained after decellularization had been operated (Yagi et al., 2013). But firstly, recellularization is not as easy as decellularization. After sequentially perfusion of the organ scaffold, it had been found that the cells were only concentrated around the perfusion vessels, which was completely different from the uniform dispersion of the cells in the tissue as hypothesized (Caires-Júnior et al., 2021). Apart from that, another problem is construction of functional vascular network which can transport nutrients and take away metabolic waste (Devalliere et al., 2018). Although re-endothelialization offers a non-thrombogenic surface (Baptista et al., 2011), it is extremely difficult to achieve re-endothelialization of human-scale whole-organ vessels *ex vivo*. For organ-scale scaffolds, it is necessary to construct a complete vascular system. But for vessels, is it necessary to reconstruct vascular endothelium before transplantation? The mean endothelial coverage rate in the experiment was 14% ± 8%. Despite this, the patency rate reached 83% and remained stable for at least 1 year, which may be attributed to the anticoagulant treatment. (Dahl et al., 2011). Furthermore, various surface modifications have also obtained considerable experimental data in the absence of endothelialisation *in vitro*. Whole blood preconditioning and surface modification not only spent less time *in vitro*, but also maintained a higher patency rate *in vivo*. Then this innovation can also be reproduced in the process of recellularization of decellularized parenchymal organ scaffolds (Bao et al., 2015). In the future, the development of rapid and ECM-protective decellularization methods with effective *in vitro* preconditioning will bring about a paradigm shift in the field of xenotransplantation.
